# A prediction model for distant metastasis after isolated locoregional recurrence of breast cancer

**DOI:** 10.1007/s10549-023-06901-7

**Published:** 2023-03-04

**Authors:** Takeshi Murata, Masayuki Yoshida, Sho Shiino, Ayumi Ogawa, Chikashi Watase, Kaishi Satomi, Kenjiro Jimbo, Akiko Maeshima, Eriko Iwamoto, Shin Takayama, Akihiko Suto

**Affiliations:** 1 Department of Breast Surgery, National Cancer Center Hospital, 5-1-1 Tsukiji, Chuo-Ku, Tokyo, 104-0045 Japan; 2 Department of Diagnostic Pathology, National Cancer Center Hospital, 5-1-1 Tsukiji, Chuo-Ku, Tokyo, 104-0045 Japan

**Keywords:** Breast cancer, Isolated locoregional recurrence, Chest wall recurrence, Ipsilateral breast tumor recurrence, Progesterone receptor status, Distant metastasis

## Abstract

**Purpose:**

The impact of progesterone receptor (PR) status on the prognosis of breast cancer after isolated locoregional recurrence (ILRR) remains unclear. This study evaluated the impact of clinicopathologic factors, including PR status of ILRR, on distant metastasis (DM) after ILRR.

**Methods:**

We retrospectively identified 306 patients with ILRR diagnosed at the National Cancer Center Hospital between 1993 and 2021 from the database. Cox proportional hazards analysis was performed to examine factors associated with DM after ILRR. We developed a risk prediction model based on the number of detected risk factors and estimated survival curves using the Kaplan–Meier method.

**Results:**

During a median follow-up time of 4.7 years after ILRR diagnosis, 86 patients developed DM, and 50 died. Multivariate analysis revealed that seven risk factors were associated with poor distant metastasis-free survival (DMFS): estrogen receptor-positive/PR-negative/human epidermal growth factor receptor 2-negative ILRR, short disease-free interval, recurrence site other than ipsilateral breast, no-resection of ILRR tumor, chemotherapy for the primary tumor, nodal stage in the primary tumor, and no endocrine therapy for ILRR. The predictive model classified patients into 4 groups based on the number of risk factors: low-, intermediate-, high-, and the highest-risk groups with 0 to 1, 2, 3 to 4, and 5 to 7 factors, respectively. This revealed significant variation in DMFS among the groups. A higher number of the risk factors was associated with poorer DMFS.

**Conclusion:**

Our prediction model, which considered the ILRR receptor status, may contribute to the development of a treatment strategy for ILRR.

**Supplementary Information:**

The online version contains supplementary material available at 10.1007/s10549-023-06901-7.

## Introduction

Novel multidisciplinary approaches for the treatment of breast cancer have recently been reported [1–3]. However, the incidence of locoregional recurrence after initial breast cancer treatment remains 3–10% [1–3]. Isolated locoregional recurrence (ILRR) is associated with an increased risk of distant metastasis (DM) and death [3–6]. Distant metastasis-free survival (DMFS) and overall survival (OS) of patients with breast cancer who develop ILRR are associated with age at diagnosis, type of surgery performed for primary breast cancer, tumor size, nodal metastasis, hormone receptor status of the primary tumor, disease-free interval (DFI), and ILRR site [7–11]. Although a risk stratification system using some of these factors (lymph node metastasis, a DFI < 30 months, and regional recurrence as the ILRR type) for subsequent DM and death following ILRR has been proposed [9], a prognostic model considering ILRR receptor status has not yet been developed. As shown in two prospective studies [12, 13], ILRR receptor status is important when considering treatment strategies for ILRR. Although the SAKK 23/82 trial revealed that tamoxifen improved the disease-free survival (DFS) of estrogen receptor (ER)-positive ILRR [12] and the CALOR trial demonstrated that chemotherapy benefitted patients with resected ER-negative ILRR and did not support the use of chemotherapy for those with ER-positive ILRR [13], progesterone receptor (PR) status and human epidermal growth factor receptor 2 (HER2) status of ILRR have not been fully investigated. Additional analysis of the CALOR study suggests that the prognosis of ER-positive ILRR tumors may differ depending on the PR status of ILRR [14]. A prognostic model that considers not only the ER status but also the PR and HER2 status of ILRR may be useful in deciding treatment strategies for patients with ILRR. We investigated risk factors for DM after ILRR diagnosis and developed a model to predict the probability of DM after ILRR that considers its receptor status.

## Patients and methods

### Patient characteristics

A total of 306 patients with ILRR diagnosed with primary or recurrent breast cancer at the National Cancer Center Hospital between January 1993 and December 2021 were identified. The inclusion criteria were: (1) diagnosis of primary or recurrent breast cancer at the National Cancer Center Hospital between 1993 and 2021 and (2) ILRR as the first recurrence. The exclusion criteria were: (1) bilateral breast cancer, (2) stage IV disease at initial diagnosis, (3) inflammatory breast cancer, (4) male breast cancer, (5) distant recurrence, and (6) concomitant distant recurrence and LRR as the first recurrence. Metastatic diseases, concurrent with the diagnosis of ILRR, were excluded by computed tomography or positron emission tomography/computed tomography scans. Patients with distant recurrence within 3 months of ILRR diagnosis were also excluded because the possibility of simultaneous recurrence with LRR could not be excluded. All patients with ILRR were diagnosed using core needle biopsy (CNB) or resection or fine needle aspiration cytology (FNA). The medical records of the included patients were procured from our prospectively generated database to obtain patient age at initial diagnosis, primary tumor size, primary nodal status, histological grade (HG), ER status, PR status, HER2 status, presence or absence of lymphovascular invasion (LVI), type of initial surgery, chemotherapy (CT), postoperative radiotherapy (RT), endocrine therapy (ET), location of recurrent tumor, local and systemic therapy after ILRR. We obtained ER, PR, and HER2 status at the time of initial surgery for primary breast cancer and at the time of CNB for recurrent tumors or ILRR resection. Local recurrence was defined as the presence of a tumor in the ipsilateral breast after initial breast-conserving surgery (BCS) or the presence of a tumor in the chest wall (CW)/skin after initial mastectomy. Regional recurrence was defined as the presence of tumors in the regional lymph nodes, such as the internal mammary, supraclavicular, infraclavicular, or ipsilateral axillary nodes. Surgery for local recurrent tumors was defined as salvage mastectomy for ipsilateral breast tumor recurrence (IBTR) and resection for CW/skin recurrence. The resection for axillary lymph node recurrence was complete level I and II axillary lymph node dissection (ALND) after a prior sentinel node biopsy (SLNB) or resection of the recurrent tumor after a prior complete ALND.

ER and PR were considered positive if the immunohistochemistry staining was positive in more than 1% of tumor cells [15]. A HER2 positive result corresponded to a score of 3 + on immunohistochemistry or amplification on fluorescence in situ hybridization [16]. The TNM staging of breast cancer was based on the eighth edition of the American Joint Committee on Cancer staging manual [17]. DFI was defined as the time from the initial surgery to the first detection of ILRR event(s). DMFS after ILRR was defined as the time from the diagnosis of ILRR to the first incidence of DM or death from any cause.

### Statistical analyses

The Cox proportional hazard model was used to evaluate the independent prognostic effects of risk factors on DMFS after ILRR. Baseline variables (*p* < 0.05) in the univariate analysis were included in the multivariable analysis. For cases with unknown receptor status of the ILRR tumor, the receptor status of the primary tumor was used as a proxy for analysis. We developed a risk prediction model using variables associated with DMFS using multivariate analysis (*p* < 0.10) to predict the probability of DM after ILRR. Survival curves were estimated using the Kaplan–Meier method and survival estimates were compared using the log-rank test. All statistical analyses were conducted using the statistical software, STATA SE version 16 (StataCorp LP, College Station, TX). *p* < 0.05 was set as the threshold for significance.

## Results

### Patient characteristics

Patient characteristics are summarized in Table 1. The median follow-up time after ILRR diagnosis was 4.7 years (interquartile range: 2.3–7.1). During the follow-up period, 86 patients (28.1%) had DM after ILRR, and 50 (16.3%) died. Of the patients who died, 46 died from breast cancer and the remaining four died from causes other than breast cancer. Among the 306 patients, 125 had only IBTR, 58 had only CW recurrence, 74 had only axillary node recurrence, and 22 had other regional node recurrences. Twenty-seven patients had regional node (RN) recurrence with concomitant IBTR (14 patients) or CW recurrence (13 patients). Among 139 patients with IBTR, 100 patients developed recurrent tumors in the same quadrant as the primary tumor, while in the remaining 39 patients, recurrent tumors developed in a different quadrant. Recurrent tumors were ER-positive/PR-positive/HER2-negative in 154 patients, ER-positive/PR-negative/HER2-negative in 52 patients, HER2-positive (irrespective of ER and PR status) in 33 patients, and ER-negative/PR-negative/HER2-negative in 31 patients. Thirty six patients had unknown receptor status of ILRR tumor. This was because performing CNB or ILRR resection was difficult and tissue samples for staining could not be obtained. Among these 36 patients, primary tumors were ER-positive/PR-positive/HER2-negative in 16 patients, ER-positive/PR-negative/HER2-negative in 10 patients, HER2-positive tumors (irrespective of ER and PR status) in 5 patients, and ER-negative/PR-negative/HER2-negative in 5 patients. In some patients, HG and receptor status of the primary tumor were unknown. This was because the initial surgery was performed at other hospitals and information on the primary tumor was not available or because evaluation of receptor status was not performed or was incomplete at the time of patient transfer or surgery at our hospital. Among the 306 patients, 247 underwent surgery for ILRR. Regarding the first DM site after ILRR, lung, liver, bone, distant lymph node, brain, and other sites were observed in 28, 15, 19, 15, 5, and 4 patients, respectively (Table 1).

**Table 1:** Baseline characteristics

Variables	*N*	(%)
Age (y) (at primary breast cancer)		
< 50	152	(49.7)
≥ 50	154	(50.3)
ILRR site		
Ipsilateral breast	125	(40.9)
Chest wall	58	(19.0)
Regional node	96	(31.4)
Ipsilateral breast with regional node	14	(4.6)
Chest wall with regional node	13	(4.3)
DFI (Months)		
< 24	59	(19.3)
24–48	67	(21.9)
≥ 48	180	(58.8)
ER status (at ILRR)		
Positive	223	(72.9)
Negative	47	(15.4)
Unknown	36	(11.8)
PR status (at ILRR)		
Positive	166	(54.3)
Negative	104	(34.0)
Unknown	36	(11.8)
HER2 status (at ILRR)		
Positive	33	(10.8)
Negative	237	(77.5)
Unknown	36	(11.8)
Receptor status (at ILRR)		
ER + /PR + /HER2-	154	(50.3)
ER + /PR-/HER2-	52	(17.0)
HER2 + (irrespective of ER and PR status)	33	(10.8)
ER-/PR-/HER2-	31	(10.1)
Unknown	36	(11.8)
Ki-67 (at ILRR)		
< 20%	63	(20.6)
≥ 20%	113	(36.9)
Unknown	130	(42.5)
Resection (at ILRR)		
Yes	247	(80.7)
No	59	(19.3)
RT (at ILRR)		
Chest wall	30	(9.8)
Regional node	33	(10.8)
Chest wall + Regional node	29	(9.5)
No	214	(69.9)
CT (at ILRR)		
Yes	125	(40.9)
No	179	(58.5)
Unknown	2	(0.7)
Anti-HER2 therapy (at ILRR)		
Yes	25	(8.2)
No	281	(91.8)
ET (at ILRR)		
Yes	214	(69.9)
No	90	(29.4)
Unknown	2	(0.7)
First DM site after ILRR		
Lung	28	(9.2)
Liver	15	(4.9)
Bone	19	(6.2)
Distant lymph nodes	15	(4.9)
Brain	5	(1.6)
Others	4	(1.3)
No distant metastasis	220	(71.9)
Tumor stage (primary)		
Tis	24	(7.8)
T1	129	(42.2)
T2	114	(37.3)
T3	29	(9.5)
Unknown	10	(3.3)
Nodal stage (primary)		
N0	192	(62.8)
N1	70	(22.9)
N2	25	(8.2)
N3	15	(4.9)
Unknown	4	(1.3)
HG (at primary)		
1	44	(14.4)
2	121	(39.5)
3	112	(36.6)
Unknown	29	(9.5)
LVI (primary)		
Positive	158	(51.6)
Negative	122	(39.9)
Unknown	26	(8.5)
ER status (primary)		
Positive	231	(75.5)
Negative	60	(19.6)
Unknown	15	(4.9)
PR status (primary)		
Positive	182	(59.5)
Negative	101	(33.0)
Unknown	23	(7.5)
HER2 status (primary)		
Positive	33	(10.8)
Negative	249	(81.4)
Unknown	24	(7.8)
Breast Surgery (primary)		
TM	125	(40.9)
BCS	181	(59.2)
Axillary surgery (primary)		
SLNB only	141	(46.1)
ALND	165	(53.9)
RT (primary)		
WBI after BCS with/without RNI	154	(50.3)
Chest wall with/without RNI	19	(6.2)
No	133	(43.5)
CT (primary)		
Neoadjuvant	42	(13.7)
Adjuvant	86	(28.1)
No	178	(58.2)
Anti-HER2 therapy (primary)		
Yes	22	(7.2)
No	284	(92.8)
ET (primary)		
Yes	182	(59.5)
No	124	(40.5)

*HG* histological grade, *LVI* lymphovascular invasion, *ER* estrogen receptor, *PR* progesterone receptor, *HER2* human epidermal growth factor receptor 2, *BCS* breast-conserving surgery, *TM* total mastectomy, *RT* radiotherapy, *CT* chemotherapy, *ET* endocrine therapy, *DFI* disease-free interval, *SLNB* sentinel lymph node biopsy, *ALND* axillary lymph node dissection, *ILRR* isolated locoregional recurrence, *DM* distant metastasis, *WBI* whole breast irradiation, *RNI* regional node irradiation

### Prognostic factors for DMFS after ILRR diagnosis

In the univariate analysis of DMFS, the following were significant poor prognostic factors: older age at primary breast cancer diagnosis (hazard ratio [HR] 1.79; 95% confidence interval [CI] 1.15–2.79; *p* = 0.010), DFI shorter than 24 months (HR 4.41; 95% CI 2.65–7.34; *p* < 0.001), DFI between 24 and 48 months (HR 2.29; 95% CI 1.36–3.86; *p* = 0.002), CW recurrence (HR 3.80; 95% CI 2.05–7.05; *p* < 0.001), RN recurrence (HR 3.23; 95% CI 1.80–5.76; *p* < 0.001), CW with RN recurrence (HR 7.40; 95% CI 3.05–18.0; *p* < 0.001), ER-positive/PR-negative/HER2-negative ILRR (HR 3.31; 95% CI 2.01–5.48; *p* < 0.001) and HER2-positive ILRR (HR 2.22; 95% CI 1.13–4.35; *p* = 0.020), ER-negative/PR-negative/HER2-negative ILRR (HR 2.96; 95% CI 1.54–5.70; *p* = 0.001), non-resection of ILRR (HR 5.02; 95% CI 3.28–7.70; *p* < 0.001), no endocrine therapy administered for ILRR (HR 2.25; 95% CI 1.46–3.45; *p* < 0.001), larger tumor size at primary (HR:1.75; 95% CI 1.08–2.84; *p* = 0.022 for T2, HR 3.05; 95% CI 1.66–5.62; *p* < 0.001 for T3) and positive nodal status in the primary tumor (HR 2.01; 95% CI 0.03–3.37; *p* = 0.008 for N1, HR 5.36; 95% CI 3.01–9.51; *p* < 0.001 for N2, HR 6.89; 95% CI 3.29–14.4; *p* < 0.001 for N3), HG3 (HR 2.66; 95% CI 1.26–5.64; *p* = 0.010) and LVI in the primary tumor (HR 2.04; 95% CI 1.32–3.15; *p* = 0.001), total mastectomy for the primary tumor (HR 2.70; 95% CI 1.75–4.17, *p* < 0.001), and CT for the primary tumor (HR 3.72; 95% CI 2.37–5.83, *p* < 0.001) (Table 2).

**Table 2:** Uni- and multivariate analysis results of factors associated with distant metastasis after isolated locoregional recurrence

Factor	Category	Univariate		Multivariate	
		HR (95% CI)	*p* value	HR (95% CI)	*p* value
Age (primary)	< 50y	Reference		Reference	
	≥ 50y	1.79 (1.15–2.79)	0.010	1.08 (0.65–1.80)	0.758
ILRR site	Ipsilateral breast	Reference		Reference	
	CW	3.80 (2.05–7.05)	< 0.001	2.62 (0.97–7.05)	0.057
	RN	3.23 (1.80–5.76)	< 0.001	2.10 (0.94–4.70)	0.070
	Ipsilateral breast + RN	0.90 (0.21–3.90)	0.887	1.23 (0.24–5.39)	0.881
	CW + RN	7.40 (3.05–18.0)	< 0.001	6.19 (1.82–21.1)	0.004
DFI (Months)	≥ 48	Reference		Reference	
	24–48	2.29 (1.36–3.86)	0.002	2.18 (1.21–3.93)	0.009
	< 24	4.41 (2.65–7.34)	< 0.001	2.27 (1.09–4.73)	0.028
Receptor status (ILRR)	ER + /PR + /HER2-	Reference		Reference	
	ER + /PR-/HER2-	3.31 (2.01–5.48)	< 0.001	2.37 (1.33–4.24)	0.004
	HER2 +	2.22 (1.13–4.35)	0.020	0.75 (0.30–1.87)	0.532
	ER-/PR-/HER2-	2.96 (1.54–5.70)	0.001	0.78 (0.33–1.89)	0.587
Resection (ILRR)	Yes	Reference		Reference	
	No	5.02 (3.28–7.70)	< 0.001	1.93 (1.06–3.52)	0.032
RT (ILRR)	No	Reference			
	CW	1.03 (0.52–2.02)	0.937		
	RN	1.39 (0.75–2.60)	0.297		
	CW and RN	1.01 (0.46–2.22)	0.975		
CT (ILRR)	No	Reference			
	Yes	1.03 (0.67–1.59)	0.888		
ET (ILRR)	Yes	Reference		Reference	
	No	2.25 (1.46–3.45)	< 0.001	2.16 (1.09–4.28)	0.028
Tumor stage (primary)	T1	Reference		Reference	
	Tis	0.21 (0.03–1.51)	0.120	0.34 (0.04–2.86)	0.331
	T2	1.75 (1.08–2.84)	0.022	1.07 (0.61–1.89)	0.805
	T3	3.05 (1.66–5.62)	< 0.001	0.52 (0.23–1.15)	0.107
Nodal status (primary)	N0	Reference		Reference	
	N1	2.01 (0.03–3.37)	0.008	1.02 (0.53–1.98)	0.942
	N2	5.36 (3.01–9.51)	< 0.001	2.55 (1.14–5.70)	0.023
	N3	6.89 (3.29–14.4)	< 0.001	2.63 (0.96–7.18)	0.059
Breast Surgery (primary)	BCS	Reference		Reference	
	TM	2.70 (1.75–4.17)	< 0.001	0.62 (0.31–1.26)	0.186
HG (primary)	HG1	Reference		Reference	
	HG2	1.12 (0.51–2.47)	0.775	0.56 (0.23–1.36)	0.197
	HG3	2.66 (1.26–5.64)	0.010	0.82 (0.34–1.97)	0.650
LVI (primary)	No	Reference		Reference	
	Yes	2.04 (1.32–3.15)	0.001	1.56 (0.90–2.71)	0.115
CT (primary)	No	Reference		Reference	
	Yes	3.72 (2.37–5.83)	< 0.001	2.20 (1.24–3.89)	0.007
ET (primary)	No	Reference			
	Yes	1.10 (0.71–1.71)	0.655		
RT (primary)	No	Reference			
	Yes	0.80 (0.52–1.22)	0.291		

*ER* estrogen receptor, *PR* progesterone receptor, *HER2* human epidermal growth factor receptor 2, *ILRR* isolated locoregional recurrence, *DFI* disease-free interval, *RT* radiotherapy, *CT* chemotherapy, *ET* endocrine therapy, *HG* histologic grade, *LVI* lymphovascular invasion, *TM* total mastectomy, *BCS* Breast-conserving surgery, *CW* chest wall, *RN* regional node, *HR* hazard ratio, *CI* confidence interval

In the multivariate analysis the following were significant poor prognostic factors: DFI shorter than 24 months (HR 2.27; 95% CI 1.09–4.73; *p* = 0.028), DFI between 24 and 48 months (HR 2.18; 95% CI 1.21–3.93; *p* = 0.009), CW with RN recurrence (HR 6.19; 95% CI 1.82–21.1; *p* = 0.004), ER-positive/PR-negative/HER2-negative ILRR (HR 2.37; 95% CI 1.33–4.24; *p* = 0.004), non-resection of ILRR (HR 1.93; 95% CI 1.06–3.52; *p* = 0.032), no endocrine therapy administered for ILRR (HR 2.16; 95% CI 1.09–4.28; *p* = 0.028), primary tumor N2 stage (HR 2.55; 95% CI 1.14–5.70; *p* = 0.023), and CT for the primary tumor (HR 2.20; 95% CI 1.24–3.89; *p* = 0.007) (Table 2). Isolated CW recurrence (HR 2.62; 95% CI 0.97–7.05; *p* = 0.057), isolated RN recurrence (HR 2.10; 95% CI 0.94–4.70; *p* = 0.070), and N3 stage in the primary tumor (HR 2.63; 95% CI 0.96–7.18; *p* = 0.059) tended to be poor prognostic factors (Table 2).

### DMFS stratified by the number of risk factors

**Fig. 1:**
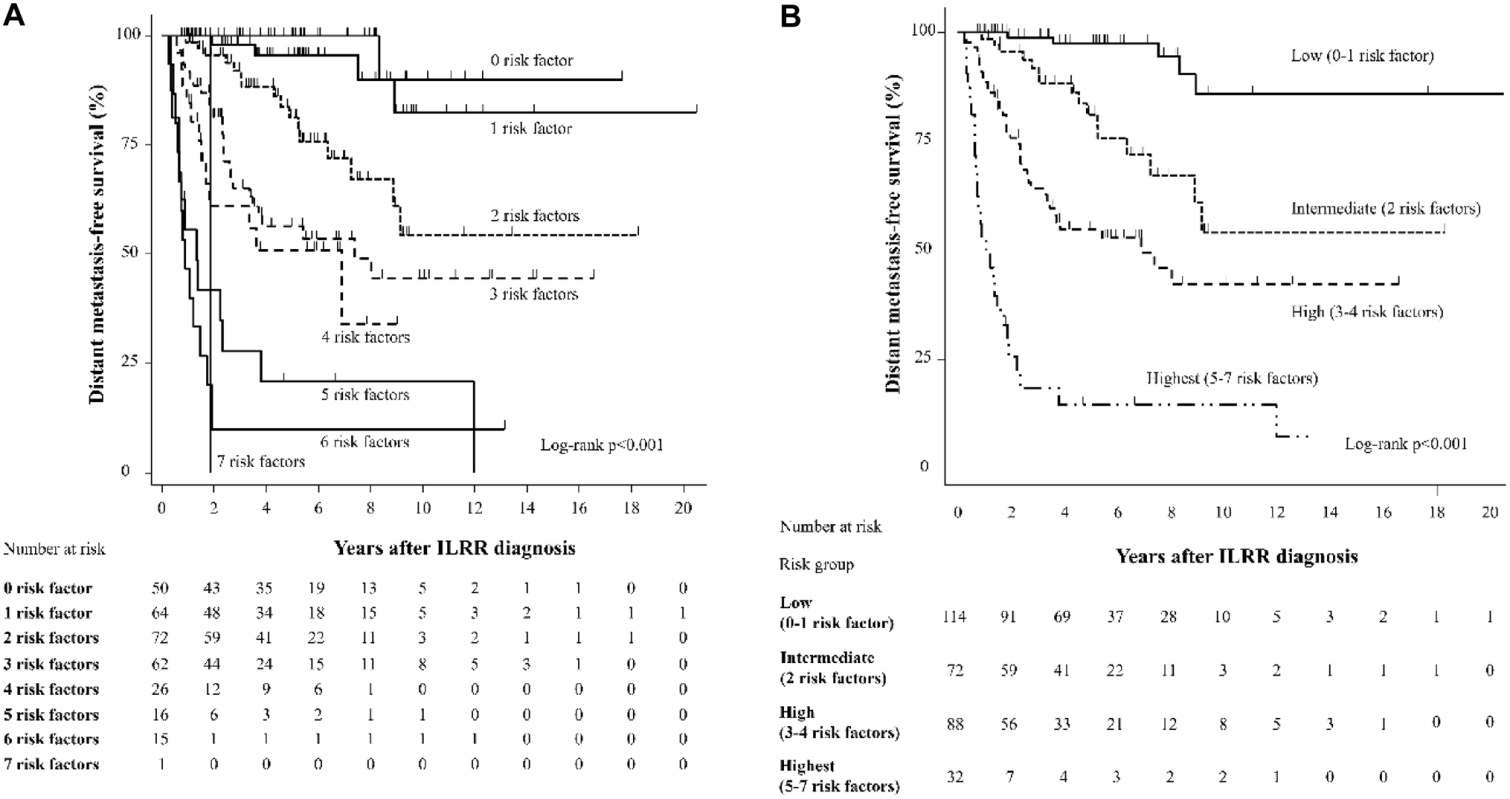
DMFS after ILRR according to the number of risk factors (**A**) and the risk groups (**B**). The risk factors were ILRR receptor status (ER-positive/PR-negative/HER2-negative tumor), shorter DFI (DFI shorter than 24 months or DFI between 24 and 48 months), recurrence site (chest wall with or without regional node and isolated regional node), non-resection of ILRR, CT for the primary tumors, nodal stage at the primary tumor (N2 or N3), and no ET for the ILRR. The four risk groups were based on the number of risk factors: low risk (0 to 1 risk factors), intermediate risk (2 risk factors), high risk (3 to 4 risk factors), and highest-risk (5 to 7 risk factors) group. *DFI* disease-free interval, *DMFS* distant metastasis-free survival, *ER* estrogen receptor, *HER2* human epidermal growth factor receptor 2, *ILRR*, isolated locoregional recurrence, *PR* progesterone receptor

We developed a risk prediction model using risk factors associated with DM after ILRR. The risk factors were: ILRR receptor status (ER-positive/PR-negative/HER2-negative tumor), shorter DFI (DFI shorter than 24 months or DFI between 24 and 48 months), recurrence site (CW with or without RN and isolated RN), non-resection of ILRR, CT for the primary tumors, nodal stage at the primary tumor (N2 or N3), and no ET for the ILRR. When the risk of distant metastasis after ILRR was stratified by the number of risk factors, patients with a higher number of risk factors had significantly poorer DMFS (Fig. 1A, Supplemental Table 1). For convenience in routine clinical practice, we then classified all 306 patients into four groups based on the number of risk factors present: low risk (0–1 risk factors), intermediate risk (2 risk factors), high risk (3–4 risk factors), and highest-risk (5–7 risk factors) group. DMFS also significantly varied among the four groups (Fig. 1B). A higher number of risk factors was associated with poorer 3-year DMFS: 98.9%, low-risk group; 92.0%, intermediate-risk group; 64.2%, high-risk group; 18.3%, highest-risk group (Table 3). The distribution of each risk factor in the four risk groups is presented in Table 4. In the low-risk group, the most common risk factor was recurrence site (CW with or without RN, or isolated RN) and none of the patients had non-resection of ILRR tumor or nodal stage at primary tumor (N2 or N3) as a risk factor. In the intermediate-risk group the most common risk factor was recurrence site, followed by DFI (DFI shorter than 24 months or DFI between 24 and 48 months). Similar to the low-risk and intermediate-risk groups, recurrence site represented the most common risk factor in the high-risk group, followed by CT for the primary tumors, and then DFI (shorter than 24 months or between 24 and 48 months. Finally, in the highest risk group non-resection of ILRR tumors was the most common risk factor in addition to recurrence sites, CT for the primary tumor and DFI.

**Table 3:** Hazard ratios of the risk prediction model for DMFS after ILRR

Risk group	3-Year DMFS (95% CI)	Adjusted HR^a^ (95% CI)	*p* value
Low (0—1 risk factors) (n = 114)	98.9% (92.6–99.9)	Reference	
Intermediate (2 risk factors) (n = 72)	92.0% (81.8–96.6)	4.48 (1.60–12.6)	0.004
High (3—4 risk factors) (n = 88)	64.2% (52.3–73.8)	10.7 (4.09–28.1)	< 0.001
Highest (5—7 risk factors) (n = 32)	18.3% (6.9–34.1)	41.9 (14.8–118.8)	< 0.001
Total (n = 306)	78.6% (73.2–83.0)		

The risk factors were ILRR receptor status (ER-positive/PR-negative/HER2-negative tumor), shorter DFI (DFI shorter than 24 months or DFI between 24 and 48 months), recurrence site (chest wall with or without regional node and isolated regional node), non-resection of ILRR, CT for the primary tumors, nodal stage at the primary tumor (N2 or N3), and no ET for the ILRR. The four risk groups were based on the number of risk factors: low risk (0 to 1 risk factors), intermediate risk (2 risk factors), high risk (3 to 4 risk factors), and highest-risk (5 to 7 risk factors) group

*CI* confidence interval, *DFI* disease-free interval, *DMFS* distant metastasis-free survival, *ER*, estrogen receptor, *HER2* human epidermal growth factor receptor 2; HR, hazard ratio; ILRR, isolated locoregional recurrence; PR, progesterone receptor

^a^Adjusted by age at primary breast cancer diagnosis, primary tumor size, histologic grade of the primary tumor, lymphovascular invasion status of the primary tumor

**Table 4:** Distribution of each risk factor among the four risk groups

	a	b	Risk factor						
			Receptor status	DFI	Recurrence site	Resection of ILRR	CT for the primary tumor	Nodal stage at the primary tumor	ET for the ILRR
			ER + /PR-/HER2-	< 24 m or 24 m–48 m	CW ± RN or isolated RN	No	Yes	N2-N3	No
Risk groups			n (%)	n (%)	n (%)	n (%)	n (%)	n (%)	n (%)
Low	0	50	0 (0)	0 (0)	0 (0)	0 (0)	0 (0)	0 (0)	0 (0)
(n = 114)	1	64	6 (9)	11 (17)	21 (33)	0 (0)	14 (22)	0 (0)	12 (19)
Intermediate (n = 72)	2	72	16 (22)	30 (42)	42 (58)	5 (7)	26 (36)	3 (4)	22 (31)
High	3	62	19 (31)	33 (53)	50 (81)	13 (21)	38 (61)	10 (16)	23 (37)
(n = 88)	4	26	9 (35)	21 (81)	24 (92)	11 (42)	21 (81)	7 (27)	9 (35)
Highest	5	16	7 (44)	15 (84)	15 (94)	14 (88)	15 (94)	4 (25)	10 (63)
(n = 32)	6	15	4 (27)	15 (100)	15 (100)	15 (100)	15 (100)	12 (80)	13 (87)
	7	1	1 (100)	1 (100)	1 (100)	1 (100)	1 (100)	1 (100)	1 (100)

^a^Number of the risk factor

^b^Number of the patient

*CT* chemotherapy, *CW* chest wall, *DFI* disease-free interval, *ER* estrogen receptor, *ET* endocrine therapy, *HER2* human epidermal growth factor receptor 2, *ILRR* isolated locoregional recurrence, *m* months, *PR* progesterone receptor, *RN* regional node

## Discussion

We investigated the risk factors for DM after ILRR diagnosis and developed a model to predict the probability of DM after ILRR. Seven prognostic factors associated with poor DMFS after ILRR among patients with breast cancer were identified: ILRR receptor status (ER-positive/PR-negative/HER2-negative tumor), shorter DFI (DFI shorter than 48 months), recurrence site (chest wall recurrence with or without regional node, and isolated regional node recurrence), non-resection of ILRR, nodal stage in the primary tumor (N2 or N3), CT for the primary tumor, and no ET for the ILRR. A higher number of risk factors was associated with poorer DMFS. To our knowledge, this is the first report of a prediction model for evaluating the probability of DM after ILRR that considers the receptor status of the ILRR.

Young age at diagnosis, large tumor size, nodal involvement, a short DFI, non-IBTR ILRR, mastectomy for the primary tumor, and hormone receptor negativity of primary tumors have been shown to be adverse prognostic factors after ILRR [7–11]. In our study, short DFI, nodal involvement of the primary tumor, and the chest wall with regional nodal recurrence were significant risk factors for DM after ILRR; isolated CW recurrence and isolated RN recurrence were marginal risk factors for DM after ILRR. Furthermore, we observed that ER-positive/PR-negative/HER2-negative ILRR was a significant risk factor for poor DMFS.

PR-negativity in primary tumors and PR-negativity in recurrent tumors (including distant metastases) are poor prognostic factors [18–22]. We demonstrated that ER-positive/PR-negative/HER2-negative ILRR was a poor prognostic factor. This result was consistent with the findings of a previously reported trial [14]. Although the number of patients with PR-negative tumors was small in the CALOR trial, the proportion of all subsequent DFS events after an ILRR was higher in the ER-positive/PR-negative (15/28; 54%) than ER-positive/PR-positive (15/73; 21%) subgroups, and the proportion of cases of distant recurrence after ILRR was also higher in the ER-positive/PR-negative (8/28; 29%) than in the ER-positive/PR-positive (11/73; 15%) subgroups [14].

Conversely, for PR-negative tumors, ER-negative/PR-negative/HER2-negative tumors were not a poor prognostic factor in our study. One possible explanation for this may be the difference in the proportion of patients undergoing CT for ILRR.

Twenty-six (41.9%) and 24 (66.7%) patients underwent CT for ILRR in the ER-positive/PR-negative/HRE2-negative and ER-negative/PR-negative/HER2-negative tumor groups, respectively. Of these, 10 (38.5%) and 11 (45.2%) developed DM, respectively. Among patients who did not undergo CT, 21 (58.3%) and 2 (18.2%) patients developed DM, respectively. Therefore, DM was more common in patients with ER-positive/PR-negative/HER2-negative tumors who did not undergo CT for ILRR. Although this is a retrospective study, it is possible that differences in the rate of CT for ILRR may have influenced the results.

Contrary to previous reports [23, 24], a younger age at diagnosis of primary breast cancer was not associated with poor prognosis in terms of DMFS in our study. This finding may be explained by the fact that compared with patients diagnosed at 50 years of age or older, CT rates for ILRR were higher (46.1% vs. 35.7%) and ILRR resection rates were higher (86.8% vs. 74.7%) among patients diagnosed with primary breast cancer under the age of 50 years. These results indicated that more radical local and systemic therapies were performed in younger patients. Another possible explanation was that the proportion of IBTR ILRRs, which are considered to have a better prognosis compared to non-IBTR ILRRs [25] was higher in patients under 50 years compared to patients over 50 years (73.7% vs. 37.0%).

In contrast with previous studies [7, 9, 10], size of the primary tumor was not a significant risk factor for DM after ILRR in multivariate analysis in our study. Previous reports did not evaluate the association between ILRR receptor status and primary tumor size. Our results suggest that ILRR receptor status may be a more important factor than primary tumor size for predicting DM after ILRR.

One possible reason why the initial type of breast surgery was not a risk factor for DM after ILRR could be that local recurrence was classified as IBTR or CW recurrence. Recurrence sites after initial BCS were IBTR with or without RN in 138 (76.2%) cases, skin/chest wallCW/skin recurrence with or without RN in 3 (1.7%) cases, and isolated RN in 40 (22.1%) cases. Recurrence sites after initial mastectomy were CW recurrence with or without RN in 69 (55.2%) cases, and isolated RN in 56 (44.8%) cases. The detailed classification and analysis of the recurrence site may have weakened the effect of the initial type of surgery on the prediction of DM. In our study, CW with RN recurrence after mastectomy was a significant DM risk factor, and isolated CW recurrence was a marginal DM risk factor.

CT for the primary tumor was a significant risk factor for DM after ILRR in our study. The incidence of ILRR after CT for primary breast cancer suggested that ILRR was highly resistant to CT, and such aggressive tumors would also have a higher risk for DM after ILRR. Conversely, CT for ILRR was not a significant risk factor in univariate analysis. However, our study was retrospective and the benefit of CT for ILRR should be corroborated in prospective studies. ET for ILRR was a significant DM risk-reducing factor. The predictive accuracy of this model for DM by ILRR receptor status was equally good for all receptor statuses (Supplementary Fig. 1).

The strength of the present study is that ILRR receptor status rather than the receptor status of the primary tumor was used to develop the predictive model for DM after ILRR. Several studies suggested that PR status is an important factor in predicting breast cancer prognosis. The loss of PR expression was reportedly associated with resistance to ET, cell migration, and metastasis [26–33]. To our knowledge, this was the first study that examined the association between PR status of ILRR tumor and prognosis after ILRR. Although the CALOR trial showed no benefit of chemotherapy in patients with ER-positive ILRR, it did not evaluate the PR status of the recurrent tumor [13]. Several studies found that ER-positive/PR-negative tumors had worse breast cancer-specific survival than ER-positive/PR-positive breast cancer [34, 35] and was associated with endocrine resistance [28, 36]. Our study showed that patients with ER-positive/PR-negative/HER2-negative ILRR had significantly worse prognoses than patients with ER-positive/PR-positive/HER2-negative ILRR. This may contribute to the decision-making process regarding treatment strategies for ER-positive ILRR. Furthermore, ER-positive/PR-negative/HER2-negative tumors have been reported to be molecularly more similar to basal-like subtypes than luminal subtypes [37]. Additionally, progesterone receptor B signaling could negatively regulate breast cancer cell migration and metastasis by affecting the Cyclin-D1/Cdk4/Paxillin interaction and Paxillin phosphorylation [26]. Therefore, other treatment options such as early systemic chemotherapy, targeted therapy, and immunotherapy should be considered. The difference in estimated median progression-free survival was greater in the ER-positive/PR-negative/HER2-negative tumor than in the ER-positive/PR-positive/HER2-negative tumor when CDK4/6 inhibitor was used for patients with advanced breast cancer (9.2 months vs 7.9 months) [38].

In our study, there were 36 patients (11.8%) with unknown receptor status of ILRR, and we analyzed these patients using the receptor status of the primary tumor as a proxy. Receptor status of primary tumors and recurrent tumors are known to be discordant in a certain percentage of patients [39]. In our study ER, PR, and HER2 statuses were found to be discordant in 12.5%, 25.9%, and 7.7% of patients, respectively. Therefore, we cannot exclude the possibility that some of the patients for whom we used primary tumor receptor status as a proxy for ILRR receptor status may have had a different receptor status from the actual ILRR receptor status. This point needs to be further investigated in a large-scale study. However, in actual clinical practice, it is difficult to perform CNB or resection of ILRR in some patients and the ILRR receptor status is unknown. The risk categories of the 36 patients with unknown ILRR receptor status in this study were intermediate, high, and highest in 2, 17, and 17 patients, respectively, and DM after ILRR occurred in 0 (0%), 7 (41.2%), and 15 (88.2%) patients, respectively. These results suggest that this prediction model was useful to predict DM after ILRR risk even in patients with unknown ILRR receptor status by substituting them with primary tumor receptor status.

The present study had several limitations. First, it was a retrospective study performed at a single institution. Furthermore, the number of patients with ILRR was not large, and the follow-up period after ILRR diagnosis was relatively short. Additionally, we did not evaluate the impact of the discordance in receptor status on DM after ILRR because the receptor status of the primary tumor or recurrent tumor was unknown in a relatively large number of patients (60 of 306, 19.6%). We also did not evaluate the impact of Ki-67 value because of insufficient data. Finally, external validation is required to evaluate the feasibility of our scoring system.

## Conclusions

We investigated risk factors for DM after ILRR diagnosis and developed a prediction model to evaluate the probability of DM after ILRR. Our model based on 7 risk factors that also takes into account the tumor receptor status of ILRR, may be a useful tool in determining treatment strategies for ILRR.

## Supplementary Information

Below is the link to the electronic supplementary material.

Supplementary file1 (DOCX 195 kb)

Supplementary file2 (DOCX 20 kb)

## Data Availability

The datasets analyzed during the current study are available from the corresponding author on reasonable request.
