# Supplementary material for: A prediction model for distant metastasis after isolated locoregional recurrence of breast cancer
Source: Breast Cancer Res Treat. 2023 Mar 4;199(1):57–66. doi: 10.1007/s10549-023-06901-7 (PMC10147732; doi:10.1007/s10549-023-06901-7)
Supplement: Supplementary file 1 — Supplementary file1 (DOCX 195 kb) [file 10549_2023_6901_MOESM1_ESM.docx]

**A Prediction Model for Distant Metastasis After Isolated Locoregional Recurrence of Breast Cancer**

*Breast Cancer Research and Treatment*

Takeshi Murata^1^, Masayuki Yoshida^2^, Sho Shiino^1^, Ayumi Ogawa^1^, Chikashi Watase^1^, Kaishi Satomi^2^, Kenjiro Jimbo^1^, Akiko Maeshima^2^, Eriko Iwamoto^1^, Shin Takayama^1^, Akihiko Suto^1^

Correspondence should be addressed to:

Takeshi Murata

Department of Breast Surgery, National Cancer Center Hospital, 5-1-1 Tsukiji, Chuo-ku, Tokyo 104-0045, Japan.

Telephone number: +81-3-3547-5201

Fax number:+81-3-3542-3815

E-mail: tamurata@ncc.go.jp

ORCID: 0000-0003-0942-7599


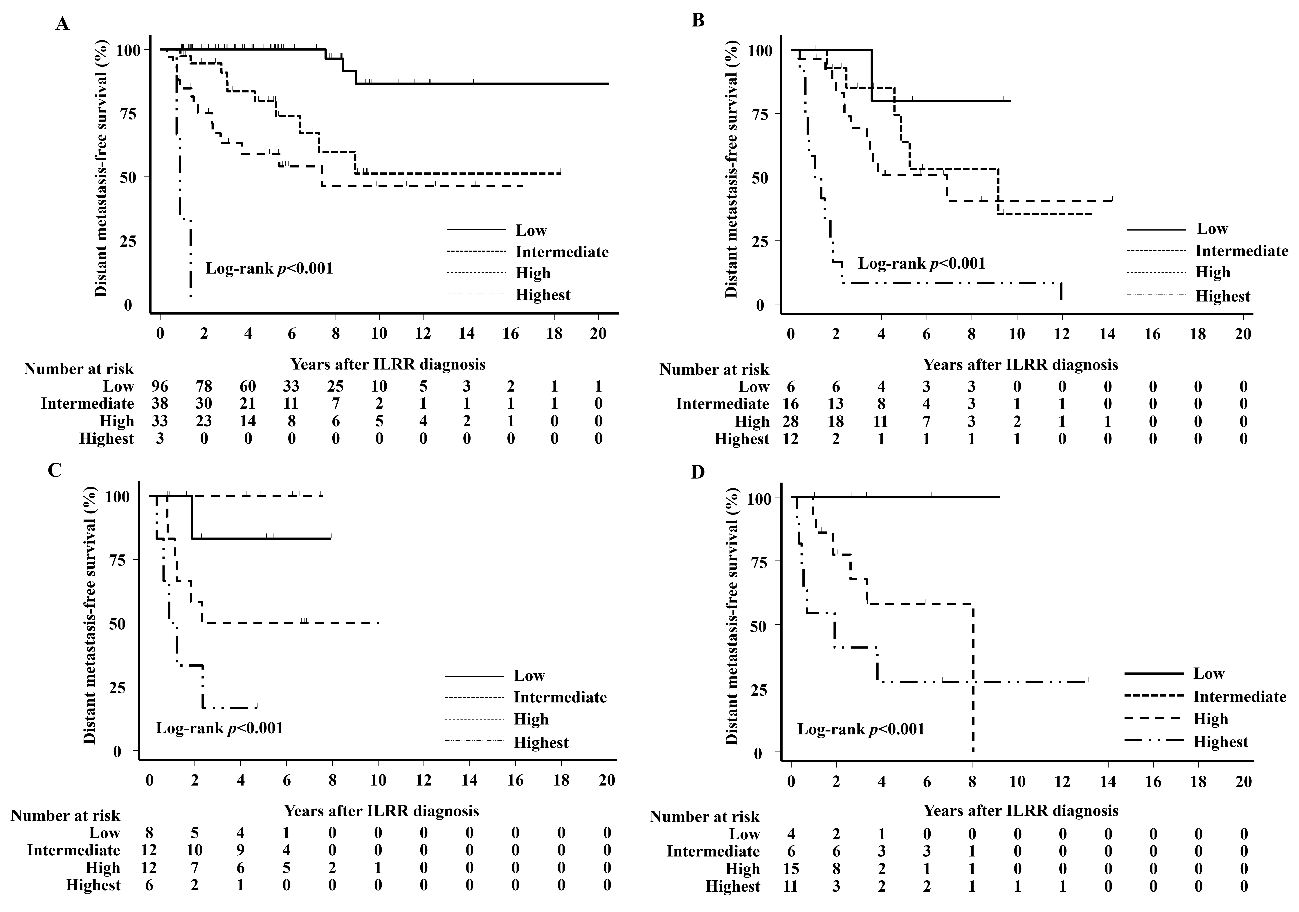


**Supplemental Figure 1:** DMFS after isolated locoregional recurrence classified using the risk prediction model according to ILRR tumor receptor status. A) ER-positive/PR-positive/HER2-negative tumor. B) ER-positive/PR-negative/HER2-negative tumor. C) HER2-positive tumor (irrespective of ER and PR status). D) ER-negative/PR-negative/HER2-negative tumor. The low-, intermediate-, high-, and the highest-risk groups had 0-1, 2, 3-4, and 5-7 risk factors, respectively. The risk factors were ILRR receptor status (ER-positive/PR-negative/HER2-negative tumor), shorter DFI (DFI shorter than 24 months or DFI between 24 and 48 months), recurrence site (chest wall with or without regional node, and isolated regional node), no-resection of ILRR, chemotherapy for the primary tumor, nodal stage in the primary tumor (N2 or N3), and no endocrine therapy administered for the ILRR.

Abbreviations: DFI, disease-free interval; DMFS, distant metastasis-free survival; ER, estrogen receptor; HER2, human epidermal growth factor receptor 2; ILRR, isolated locoregional recurrence; PR, progesterone receptor
