# Supplementary material for: A prediction model for distant metastasis after isolated locoregional recurrence of breast cancer
Source: Breast Cancer Res Treat. 2023 Mar 4;199(1):57–66. doi: 10.1007/s10549-023-06901-7 (PMC10147732; doi:10.1007/s10549-023-06901-7)
Supplement: Supplementary file 2 — Supplementary file2 (DOCX 20 kb) [file 10549_2023_6901_MOESM2_ESM.docx]

**A Prediction Model for Distant Metastasis After Isolated Locoregional Recurrence of Breast Cancer**

*Breast Cancer Research and Treatment*

Takeshi Murata^1^, Masayuki Yoshida^2^, Sho Shiino^1^, Ayumi Ogawa^1^, Chikashi Watase^1^, Kaishi Satomi^2^, Kenjiro Jimbo^1^, Akiko Maeshima^2^, Eriko Iwamoto^1^, Shin Takayama^1^, Akihiko Suto^1^

Correspondence should be addressed to:

Takeshi Murata

Department of Breast Surgery, National Cancer Center Hospital, 5-1-1 Tsukiji, Chuo-ku, Tokyo 104-0045, Japan.

Telephone number: +81-3-3547-5201

Fax number:+81-3-3542-3815

E-mail: tamurata@ncc.go.jp

ORCID: 0000-0003-0942-7599

| Number of risk factors | 3-Year DMFS (95% CI) |
| --- | --- |
| 0 (n=50) | 100% |
| 1 (n=64) | 98.0% (86.6 – 99.7) |
| 2 (n=72) | 92.0% (81.8 – 99.7) |
| 3 (n=62) | 65.2% (50.7 – 76.3) |
| 4 (n=26) | 61.0% (37.9 – 77.6) |
| 5 (n=16) | 27.8% (8.8 – 51.0) |
| 6 (n=15) | 10.0% (7.8 – 33.5) |
| 7 (n=1) | 0% |

**Supplemental Table 1:** DMFS after isolated locoregional recurrence according to the number of risk factors classified using the risk prediction model based on ILRR tumor receptor status.

Abbreviations: DMFS, distant metastasis-free survival; CI, confidence interval
